# History of malaria control in Rwanda: implications for future elimination in Rwanda and other malaria-endemic countries

**DOI:** 10.1186/s12936-020-03407-1

**Published:** 2020-10-07

**Authors:** Corine Karema, Shawn Wen, Abigail Sidibe, Jennifer L. Smith, Roly Gosling, Emmanuel Hakizimana, Marcel Tanner, Abdisalan M. Noor, Allison Tatarsky

**Affiliations:** 1 Swiss Tropical and Public Health Institute, Basel, Switzerland; 2 University of Basel, Basel, Switzerland; 3 Malaria Elimination Initiative, Global Health Group, University of California, San Francisco, USA; 4 Malaria and Other Parasitic Diseases Division, RBC–Ministry of Health, Kigali, Rwanda; 5 Strategic Information for Response, Global Malaria Programme, World Health Organization, Geneva, Switzerland; 6 Quality and Equity Health Care, Kigali, Rwanda

**Keywords:** Rwanda, Malaria, Epidemiology, Control, History

## Abstract

**Background:**

Malaria was first reported in Rwanda in the early 1900s with significant heterogeneity and volatility in transmission over subsequent decades. Here, a comprehensive literature review of malaria transmission patterns and control strategies in Rwanda between 1900 and 2018 is presented to provide insight into successes and challenges in the country and to inform the future of malaria control in Rwanda.

**Methods:**

A systematic literature search of peer-reviewed publications (Web of Knowledge, PubMed, Google Scholar, and the World Health Organization Library (WHOLIS) and grey literature on malaria control in Rwanda between 1900 and 2019 was conducted with the following search terms: “malaria”“, “Rwanda”, “epidemiology”, “control”, “treatment”, and/or “prevention.” Reports and other relevant documents were also obtained from the Rwanda National Malaria Control Programme (NMCP). To inform this literature review and evidence synthesis, epidemiologic and intervention data were collated from NMCP and partner reports, the national routine surveillance system, and population surveys.

**Results:**

Two hundred sixty-eight peer-reviewed publications and 56 grey literature items were reviewed, and information was extracted. The history of malaria control in Rwanda is thematically described here according to five phases: 1900 to 1954 before the launch of the Global Malaria Eradication Programme (GMEP); (2) Implementation of the GMEP from 1955 to 1969; (3) Post- GMEP to 1994 Genocide; (4) the re-establishment of malaria control from 1995 to 2005, and (5) current malaria control efforts from 2006 to 2018. The review shows that Rwanda was an early adopter of tools and approaches in the early 2000s, putting the country ahead of the curve and health systems reforms created an enabling environment for an effective malaria control programme. The last two decades have seen unprecedented investments in malaria in Rwanda, resulting in significant declines in disease burden from 2000 to 2011. However, in recent years, these gains appear to have reversed with increasing cases since 2012 although the country is starting to make progress again.

**Conclusion:**

The review shows the impact and fragility of gains against malaria, even in the context of sustained health system development. Also, as shown in Rwanda, country malaria control programmes should be dynamic and adaptive to respond and address changing settings.

## Background

Malaria remains a significant public health problem, disproportionately impacting morbidity and mortality in low-resource communities worldwide. After impressive progress in malaria control seen in the first decade of the century (2000–2010), the second decade appears more challenging. From 2014 to 2017, malaria cases have been on the rise in some areas. In 2018, there were approximately 228 million cases (95% confidence interval [CI] 206–258 million) of malaria worldwide, causing more than 405 000 deaths, mainly in Africa, that account for 93% of malaria cases [1].

The last two decades have seen unprecedented investments in Rwanda’s health system strengthening and increase in external funding that has supported scale-up of critical malaria control interventions, resulting in substantial declines in disease burden [2–4]. However, since 2012, the country has been experiencing a persistent upsurge of malaria. From 2012 to 2018, malaria incidence has increased from 48 per 1000 to 403 per 1000 [5] Malaria transmission occurs year-round with peaks in April–May and November–December. Although transmission is heterogeneous in Rwanda, the entire population is considered at risk of malaria [6]. The primary *Plasmodium* species found in Rwanda is *Plasmodium falciparum,* but *Plasmodium malariae* and *Plasmodium ovale* have also been identified by molecular methods [7]. The dominant malaria vector in Rwanda is *Anopheles gambiae* sensu stricto (*s.s*.), which is highly anthropophilic and considered to be one of the most efficient vectors of malaria in the world [8]. *Anopheles funestus* and *Anopheles arabiensis* are secondary vectors in Rwanda [9].

In this paper, a comprehensive review of the evolution of malaria epidemiology and control in Rwanda is presented to help understand the potential drivers of change and to inform the future of the fight against malaria in the country. The trends, critical interventions deployed over time, and underlying health system changes that have shaped the country’s malaria response in the period 1900–2018 are documented. Factors that may have contributed to the success and challenges throughout this period are discussed.

## Methods

### Literature search

A literature search of peer-reviewed publications and grey literature (e.g. annual reports) on malaria control in Rwanda between 1900 and 2019 was conducted. To identify these publications, PubMed Web of Knowledge, Google Scholar, and the World Health Organization Library (WHOLIS) were searched for the following search terms: “Rwanda” and “malaria”, with one of the following words: “epidemiology”, “control”, “treatment”, and/or “prevention”. Additional references were identified through cross-referencing the bibliographies of these publications.

All relevant grey literature available from the Rwanda National Malaria Control Programme (NMCP) was also reviewed, including annual reports and programme evaluations. Also, relevant documents and reports from the archives of the Rwanda Ministry of Health, and evaluations and reports from implementation partners and funding agencies (e.g. Belgian Cooperation, United Nations (UN) agencies, the Global Fund to Fight AIDS, Tuberculosis, and Malaria (Global Fund) and United States Agency for International Development (USAID)/President’s Malaria Initiative (PMI projects) were reviewed from 1996 to 2018 Finally, information on underlying health system changes were identified from both grey and peer-reviewed literature and included as part of this review. Additional documents were obtained from the Prince Leopold Institute of Tropical Medicine (ITG)-Antwerp Library in Belgium. Inclusion criteria comprised any articles or documents that included Rwanda, malaria, AND one of the above keywords and were written in either French or English. Articles written in other languages were excluded. Key malaria control interventions were assembled into 5 phases based on different period related to important changes related to Global malaria strategies.

### Epidemiological data analysis

To inform this literature review and evidence synthesis, epidemiological data for 2000 to 2018 was collated from the Rwanda Health Management Information System (HMIS) managed by Rwanda’s Ministry of Health (MoH) and the World Health Organization (WHO) World malaria reports. Additional epidemiological data and malaria control intervention coverage data were collected from Rwandan population health surveys from 1992 to 2018, and Rwanda’s NMCP reports covering 2000 to 2019. Data before 1992 were extracted from publications identified in this literature review an entered into MS Excel spreadsheet. Epidemiologic evidence was synthesized and presented by the year as available.

## Results

From the literature search, 268 peer-reviewed publications and 56 grey literature items were reviewed and information was extracted (Additional file 1: Appendix 3). The history of malaria control in Rwanda is thematically described here according to five phases (Additional file 2: Appendix 1: Rwanda malaria timeline) From 1900 to 1954; before the launch of the Global Malaria Eradication Program (GMEP); (2) Implementation of the GMEP from 1955 to 1969; (3) Post-GMEP to 1994 Genocide against the Tutsi; (4) the re-establishment of malaria control from 1995 to 2005; and (5) recent malaria control efforts from 2006 to 2018.

### Period 1: 1900–1954: first documentation of malaria to the development of GMEP

Historically, malaria in Rwanda was first reported being introduced by Belgian troops passing through the region during World War I [10]. Locally known as “Kapfura” in 1917, malaria became increasingly prevalent between 1920 and 1930, with endemic malaria in the plains and epidemics in the high central plateaus [11, 12]. An increase in malaria cases was first reported around the 1920s and 1930s [12, 13].

During this period, malaria research in Rwanda was led by the Medical Laboratory of Ruanda-Urundi. In 1939, a study carried out in Musanze district found a *Plasmodium* index (PI) of 11.1%, with 52% of infections due to *P. malariae* and 48% due to *P. falciparum* [14]. Clinical data from over 2200 Rwandan copper mine workers in Pande and Kilo (Congo) showed a PI (parasite stages other than gametocytes) of 88.3% and a gametocyte index of 2.7% [15–17], Apart from treating clinical patients with quinine, no other malaria control measures were implemented until 1949, as described below.

Entomological surveys conducted in and around Butare between 1939 and 1950 [14, 18] by the Medical Laboratory identified *An. funestus* as the primary malaria vector. Two studies showed that 11% of the *An. funestus* were *Plasmodium* sporozoite positive [18].

In terms of ecological factors, the varying altitudes in Rwanda were shown to be important in malaria transmission. According to population-based surveys conducted in four sites in the Ruzizi Valley of altitudes ranging from 775 m to 2030 m between 1950 and 1951, the *Plasmodium* parasite prevalence was highest at lower altitudes (98.7% and 83.4% positivity at 775 m and 900–950 m, respectively) compared to higher altitudes (46.5% positivity at the 1500–1580 m range) while only a few *P. falciparum*, *P. malariae*, and *P. vivax* cases were detected in the 2000–2030 m range [19].

New malaria control initiatives were proposed in 1946, including strategic draining and drying out of the marshes facilitated by eucalyptus planting [20]. However, this approach was never fully implemented due to a regional famine as well as population growth, which required farming and cultivation of the marshland [12]. Instead, in 1949 before the GMEP, dichloro-diphenyl-trichloroethane (DDT) spraying campaigns began and were carried out every six months in Astrida (former Butare) from 1949 to 1951 by a private sector firm with financial support from the Indigenous Well-Being Fund (IWBF). In total, 1800 km^2^ and 100,251 huts were sprayed with DDT Geigy M50, providing coverage for 260,000 people [21]. Additionally, beginning in November 1951, about 3600 hectares of marshland surrounding Butare were treated with 5–10% Gyron Buoyant DDT powder as a form of larviciding for larval source management.

After the three-year campaign, the PI dropped by 87.1% (from 51% to 7%) in Butare while remaining high in untreated Sogwe and Kavumu in Nyanza, with PIs between 53 and 75.8% [18, 21] During the same period, the primary malaria vector *An. funestus* was successfully eliminated in Butare (Jadin [18]) while *An. gambiae* sensu lato (*s.l*.), that initially disappeared after spraying, re-emerged 6 months after the completion of DDT treatment, possibly due to DDT’s higher excito-repellency on *An. gambiae* [12]. Following these results, spraying with support of IWBF was subsequently extended to Usumbura and Bubanza in 1952, to the Shangugu territory in 1954, and the whole of Rwanda-Urundi in 1956 [13].

### Period 2: The Global Malaria Eradication Programme (GMEP) in Rwanda (1955–1969)

The approach to malaria elimination in Rwanda as part of GMEP was to use insecticides to first target zones between 1500 and 2000 m where the PI was relatively low (0.05–0.15), moving systematically to areas with a higher transmission (PI of 0.80) [13]. This strategy of quarterly DDT insecticide spraying under the leadership of the Ruanda-Urundi chiefdom (now Rwanda and Burundi) began in 1955 [12]. From 1956 to 1960, there was a steady decrease in the number of malaria cases and malaria-attributed deaths from 306,809 to 155,027 malaria cases and 1955 to 118 deaths [13] in the whole Ruanda-Urundi territory. No or minimal transmission (PI = 0–0.02) occurred at altitudes higher than 2000 m in Rwanda, indicating that malaria did continue to exhibit a spatial limit during that period [13].

In 1962, Meyus, a medical director of the Congo Malaria Control Research and Coordination Centre, developed the malaria stratification system that divided Rwanda into three malaria ecologic zones based on altitude, climate, level of transmission, and parasite prevalence. Ivorra later updated this stratification with similar malaria strata in 1982.

After Rwanda gained independence in 1962, little priority was given to malaria control due to resource constraints and lack of qualified staff [12]. The GMEP also came to an end in 1969. It is unsurprising, therefore, that this lack of investment resulted in malaria becoming the leading cause of morbidity and mortality in Rwanda starting in the 1960s and with periodic epidemics in high-altitude areas not experienced in previous years (Byumba and Ruhengeri) [22].

### Period 3: Post-GMEP (1970–1990) to the 1994 Genocide against the Tutsi

There is little documentation about malaria control in Rwanda in the 1970s. In the 1980s, with growing concern about parasite resistance to the country’s first line anti-malarial drug, chloroquine (CQ), Rwanda’s Ministry of Public Health and Social Work (MPHSW) implemented malaria treatment standardization. Besides, the MPHSW provided regular medical training on chloroquine use and instituted a drug resistance monitoring system through a specialized malaria laboratory for training and operational research. During this period, the earliest therapeutic failures of chloroquine treatment for *P. falciparum* in Rwanda were reported [23]. As resistance monitoring was scaled up, anti-malarial drug resistance was reported for chloroquine in Ruhengeri [24–27], amodiaquine (AQ) [26], and sulfadoxine/pyrimethamine (SP) [25] throughout the 1980s.

Between 1982 and 1988, malaria was the leading cause of morbidity, reaching at least 40% of all consultations among outpatients in health cents with a 15% case fatality rate. A study found 9% of malaria prevalence among over 3700 women presenting to prenatal clinics at the Kigali Hospital [28]. A cost analysis study conducted in 1982 showed that the direct cost of malaria treatment was US$1.66 for treatment, including care of outpatients and hospitalized cases in both government and private facilities, as well as self-treatment. The its indirect cost of productive time lost to malaria morbidity in adults and to care for sick children, and the cost of lifetime earnings lost through premature malaria mortality was US$6.03 (in 2020 US$ dollars), the annual malaria cases were equivalent to a mean of 3.5 days of productivity loss or 1% of the Gross Domestic Product (GDP). Thus, the per capita malaria cost equals 3.5 days of production or 1% of GDP. The average cost of each of the 1722,271 reported malaria cases in 1989 was US $11.82: US$2.58 in direct and $9.24 in indirect cost.

In 1989, the government created the National Malaria Control Programme (NMCP) to manage a coordinated national response to the disease. However, the NMCP functioned poorly due to lack of funding and the staff’s capacity, and its operations were further hindered by the 1994 genocide against the Tutsi.

The 1994 Genocide against the Tutsi was responsible for the deaths of 1 million people in Rwanda with the displacement of millions more [29]. Without functioning health system building blocks, Rwanda saw frequent epidemics of infectious diseases, especially malaria, Human Immunodeficiency Virus (HIV)/Acquired Immunodeficiency Syndrome (AIDS), and tuberculosis, resulting in a crude mortality rate of 20–35 per 10,000 people each day [29]. Furthermore, Rwanda’s under-five child mortality rates were the highest in the world, and life expectancy at birth would remain the lowest globally over the subsequent few years [29]. There was a marked rural/urban divide and inequity in child survival between maternal groups with different levels of education [30].

### Period 4: re-establishment of malaria control, 1995–2005

The year following the Genocide saw a spike in malaria cases and deaths (Additional file 3: Appendix 2: Fig. S1) [31]. The spread of malaria was further catalyzed by the massive movement of 1.4 million internally displaced persons within Rwanda from more endemic to less endemic areas and another 1.5 million refugees into neighbouring Zaire, Tanzania, and Burundi [32]. As previously seen, resistance to CQ and SP among Rwandan refugees in Zaire remained high at 79.5% and 65% clinical failures, respectively [33].

In 1995, the first malaria policies were developed and focused on malaria prevention, case management, and education of the population. In 1995, the estimated direct cost per episode of malaria was US$3.55, and the indirect cost of over US$8.49 (in 2020 US Dollars) (Rwanda National Health Account (NHA). With Rwanda’s population of roughly 8 million inhabitants, each suffering an estimated two or three episodes per year, malaria-related costs represented an enormous financial burden.

In 1996, the first mosquito nets were introduced in the private sector in Rwanda with support of the Belgian cooperation, and additional 40,000 mosquito nets were procured in 2000 for a pilot evaluation project in Kayonza District with the funding support of the Swiss Cooperation [34]. Between 1998 and 2004, severe epidemics of malaria were observed with dramatic increases in malaria-attributed maternal mortality, particularly during times of heavy rainfalls in districts affected by malaria epidemics [35]. In 2000, malaria remained the leading cause of morbidity and mortality in Rwanda. It was responsible for 54% of all inpatient deaths and 50% of all outpatient visits. However, only 43% of reported malaria cases were laboratory-confirmed [36]. Malaria was also the leading cause of death among children under five, with 35% of deaths in this age group attributed to the disease. During this same period, entomological surveys found *An. gambiae s.l.* and *An. funestus* to be the predominant vectors at three different sentinel sites [9].

In 1999, Rwanda joined the East Africa Network for Monitoring Antimalarial Treatment (EANMAT) that found a clinical failure of CQ & SP in the country exceeded the WHO cut-off point (25% of total failure), requiring a change in drug policy [37–39]. In response, Rwanda’s Ministry of Health changed its treatment policy from CQ) to AQ + SP in 2001. With the support of the Belgian technical cooperation, the NMCP procured and distributed AQ + SP to all public hospitals and health centres.

In line with the new and updated Rwanda malaria policies put in place in the early 2000s, malaria control interventions included weekly doses of CQ to pregnant women to prevent malaria, together with iron supplementation as part of standard Antenatal care (ANC). Guidelines also included targeted distribution of insecticide-treated nets (ITNs) to pregnant women and children under five through all public health centres, aiming to reach high coverage within these vulnerable populations. However, overall ITN coverage and usage remained low only 7% of households possessed at least one bed-net and 8% of children under five reported sleeping under an ITN during the 2000 national census, which increased to 17% of children under five in 2005 [40]. A cross-sectional survey of 1432 pregnant women in six Rwandan health districts of variable malaria endemicity revealed that only 13.1% of women attending antenatal clinics owned a bed net at home, and even fewer (8.3%) slept under it [41].

The government of Rwanda, with the support of partners, initiated critical health systems strengthening initiatives during this period, including community-based management of malaria, community-based health insurance, and performance-based financing. Community Healthcare Workers (CHWs) were initially recruited in Rwanda in the post-Genocide period to provide mental health counseling, among other duties. In 1995, there were about 1 CHW per 667 populations in Rwanda. As the country began to recover in the following decade (2000), Rwanda decided to continue with this strategy and expand the number and role of CHWs to include additional health services, including sensitization on malaria.

A 2004 situational analysis conducted in five health districts (Nyanza, Gitwe, Kirehe, Kibilizi, Kibogora) revealed that only 2.4% of children aged under 5 years diagnosed with malaria were correctly treated according to national malaria treatment policy (USAID 2007). In response, the NMCP, in collaboration with partners, introduced home-based management (HBM) for malaria using a new type of CHWs [42, 43].

High costs of health services presented a significant barrier to health provision in Rwanda, with prices equal to 5.4% of household’s annual income per capita on health care according to a 1998 report by the World Bank (World Bank Rwanda Poverty Note 1998). In response, the Government of Rwanda sought to increase equitable access to primary care services by establishing a highly successful community-based health insurance (CBHI) scheme called “Mutuelle de Santé”, which was initially piloted in three districts between 1999 and 2000 [44]. Under CBHI, members contributed a small annual premium of US$2.37 (in 2020 US dollars) and modest user fees of US$0.39 (in 2020 US dollars) in exchange for improved access to care at the community level, including malaria diagnosis and treatment [45, 46] Donors such as the Global Fund to Fight AIDS, Tuberculosis, and Malaria (Global Fund) covered fees for the lowest income population unable to pay, estimated to be 10% of the population in 2004 [45, 46].

The Ubudehe programme, a concept rooted in Rwanda’s culture whereby assistance is provided within communities to members that are in need and have no form of assistance, was first piloted in the Butare province in early 2001. It was officially rolled out at the national level with support from the European Commission in 2004 whereby Rwanda has invested in a stratification process that has systematically identified poor groups to enable them to access all social programmes in the country, not just health insurance.

In 2001, performance-based financing (PBF), a health system innovation complementary to CBHI, was introduced to incentivize government health workers at government-run facilities to deliver better care by supplementing their salaries if a set of quantitative and qualitative health service delivery targets were met, as determined by peer evaluators and Ministry of Health personnel [47].

### Period 5: recent malaria response in Rwanda, 2006–2018

The Rwanda Demographic and Health Survey (DHS) also documented a two-fold decline of malaria prevalence among children under five from 2005 to 2010 [48]. In 2012, a cross-sectional study carried out in six districts located in three malaria transmission zones (low, medium, and high) in Rwanda found a 5% prevalence of malaria among pregnant women by polymerase chain reaction (PCR) test. The study also found that, among women who did not sleep under an ITN the previous night, 8.1% tested positive compared with 4.8% who slept under an ITN [49].

Since 2005, the Ministry of Health has developed two malaria strategic plans (2008–2012 and 2013–2018) that have set out the vision and approach to malaria control in Rwanda informed by programme reviews in 2007 and 2011 [50]. Since 2005, the main changes in malaria control interventions were scaling up of long-lasting insecticidal nets (LLINs) and artemisinin-based combination therapy (ACT) (targeting universal coverage, supplemented by indoor residual spraying (IRS) in high malaria endemic districts. From 2006 to 2018, nearly 22 million LLINs were distributed to children under five and pregnant women as part of the routine distribution (antenatal care and Expanded Programme of Immunization) and integrated through vaccination and mass household campaigns.

While some limited IRS activities were carried out in Rwanda during the GMEP as described above, large-scale spraying only began in 2007 with pyrethroid insecticides in 5 high malaria endemic districts. Rwanda was one of the first countries in Africa to adopt a strategy for Integrated Vector Management (IVM) in 2011 [51], with the IRS and LLINs as the cornerstone vector control methods. With support from the President’s Malaria Initiative (PMI), the NMCP has carried out entomological monitoring to track insecticide resistance among populations of malaria vectors since 2007 [9]. Detection of pyrethroid resistance in 2012 has guided the NMCP to shift from using pyrethroid insecticides for IRS to carbamate insecticides in 2014 and organophosphates in 2016 [52] as per the Rwanda National Strategic Plan for Insecticide Resistance Management in Malaria Vectors 2013–2017 that includes rotation of insecticides every 2 years. In 2015, The NMCP undertook a larviciding pilot using a bio-larvicide, *Bacillus thuringiensis israelensis* (Bti) and will look to scale up the use of Bti pending results from the study [53].

In 2006, following the results of anti-malarial drug efficacy tests carried out in Rwanda (Fanello et al. 2006) showing an increase of anti-malarial drug resistance, the country shifted from amodiaquine plus sulfadoxine-pyrimethamine (AQ + SP) to an ACT medicine, artemether-lumefantrine (AL). Following external evaluation of home-based management of fever (HBM) was carried out in 2006, showing the impact of expanding ACT at the community level [54, 55], ACT was broadened at the community level. In 2009, Rwanda adopted a policy that required parasitological confirmation of all malaria cases with microscopy at health facilities and rapid diagnostic tests (RDTs) at the community level, implemented through a new integrated Community Case Management (iCCM) approach that replaced the existing HBM strategy. Since then, Rwanda’s 1 CHW per 178 population have provided iCCM nationwide, including diagnosis and treatment of children under five for malaria using RDTs and locally packaged children’s ACT medicine formulation, PRIMO. Malaria services were incorporated into iCCM as part of the National Community Health Strategic Plan 2013–2018 [56].

In 2005, in line with WHO recommendations, Intermittent Preventative Treatment in pregnancy (IPTp) with SP was adopted as a national policy, and Rwanda instituted focused antenatal care (FANC) with a system of free ITN distribution to pregnant women, differently from the sale of LLINs through private outlets under USAID’s funding through Population Services International (PSI). The percentage of pregnant women receiving two or more doses of SP during their last pregnancy increased from 0.2% in 2005 to 17% in 2008. The intervention was discontinued in 2008, however, based on evidence showing a 65% therapeutic failure rate of SP in children, high prevalence of gene mutations for resistance to SP, and a decrease in malaria prevalence nationwide to 2.6% among children under 5 years of age [57–59]. (Given that this intervention was used in Rwanda for such a short period, trends in IPTp coverage are not very informative to evaluate the impact of this intervention on malaria in pregnancy outcomes.

The Rwanda Malaria Communication Strategy includes approaches to engage CHWs in community mobilization and malaria health education by using World Malaria Day, radio, Television, community meetings, and newspapers to communicate Information about malaria. A Knowledge, Attitudes, and Practices (KAP) survey was conducted in 2012 and showed a high level (> 83%) of knowledge among the population on critical malaria messages, similar to findings in the Rwanda Malaria Indicator Survey (MIS) in 2013. Same data were also found in the MIS 2017.

More than US$450 million was directly committed to Rwanda’s malaria control efforts from 2005 to 2018 (Additional file 3: Appendix 2: Fig. S4), with significant funding from the Global Fund and The President’s Malaria Initiative (PMI). Total malaria expenditure per capita was estimated to be US$6.06 in 2006 (in 2020 US Dollars) and 2009/2010 [60, 61]. During the same period, malaria transmission declined, and health insurance coverage expanded, thus driving down out-of-pocket expenditures from US$1.91 to US$0.24. National coverage of CBHI reached 91% in 2011–2012 (Additional file 3: Appendix 2: Fig. S3), with 12 districts having over 90% membership [62].

In 2011, a cost–benefit analysis was conducted that demonstrated that a sustained control programme in Rwanda would avert an estimated 38 million cases, saving $267 M for the country’s health system, and $547 M in household costs, which is equivalent to 7% of household income [63].

From 2008 to 2016, the Rwanda NMCP used updated HMIS data to identify districts with the highest malaria increase and monitored malaria intervention effectiveness to understand the main drivers of malaria increase in Rwanda. The more nimble and sub-national data-driven analysis has allowed the NMCP to tailor malaria control interventions and prioritize high-risk malaria transmission areas (mainly focusing on LLINs distribution in high malaria burden districts) based on limited available resources. However, despite these malaria control efforts, Rwanda has experienced an eightfold increase in reported malaria cases countrywide (in all 30 districts) between 2012 and 2016. During the same period, malaria incidence increased from 48 malaria cases per 1000 population in 2012 to 403 malaria cases per 1000 population at risk in 2016 [5, 53]. During this period, cases have increased from 567,407 in 2012 to 4794,778 in 2016 (Additional file 2: Appendix 2: Fig. S1). The 2015 Rwanda DHS also revealed that malaria prevalence among children under five increased from 1.4% in 2010 to 2.6% in 2015 [64]. Several factors are hypothesized to have contributed to this increase. This included: climatic variations (i.e. increased temperature and precipitation); delay of LLIN supply and delivery; insecticide (pyrethroid) resistance; short term (approximately 2 years) durability of LLINs; substandard LLINs (low insecticide content and physical deterioration); change of vector behaviors toward more outdoor biting; as well as failure to provide adequate funding for universal coverage of key malaria interventions on time [50]. As a response to tackle the persistent increase of malaria burden countrywide, Rwanda developed a malaria contingency plan (MCP) in 2015–2016 [65], which identified improved strategies to reduce the case burden, and these strategies were incorporated into the extended Malaria Strategic Plan for 2013-2020 that was developed in 2017. The MCP included expansion of test and treatment of adults at the community level, expansion of IRS from five to all high malaria burden districts, and strong behaviour change communication sensitization campaigns, as well as new financing mechanisms of LLINs. As a result, according to the WHO World Malaria Report (WMR) 2019, Rwanda reported slightly less than 465000 fewer cases in 2018 compared to 2017 [1].

## Discussion

This paper documents the history of malaria control in Rwanda across five historical periods from 1900 to 2018, which have implications for the future of malaria control in Rwanda and elsewhere. Malaria was first documented in Rwanda in the early 20th century [10]. Although prevalent in the 1920s and 1930s, malaria control measures were limited to the administration of quinine for the treatment of clinical malaria. Rwanda malaria control has shown to be an early adopter of many strategies that were successful in reducing malaria incidence in Rwanda, but malaria elimination was not achieved [2, 3, 13, 66]. In 1949 before the GMEP, a widespread vector control programme with DDT spraying campaigns was carried out even before the GMEP [12]. These campaigns resulted in the elimination of *An. funestus* in some areas [21] and were expanded to all of Rwanda-Burundi in 1956 [13]. During the GMEP era, Rwanda adhered to international guidelines to pursue elimination based on DDT spraying, a strategy endorsed by the 8th Global Assembly of Health in 1955 [67].

Malaria became the leading cause of morbidity and mortality from 1960 through the 1980s as little priority was given to malaria control due to resource constraints and lack of qualified staff [12]. Throughout the 1980s and early 1990s, there were no comprehensive malaria control interventions in place in Rwanda. The 1994 Genocide had a devastating impact on health services in Rwanda, with significant increases in the burden of malaria [29]. After the tragedy of the 1994 Genocide, the country’s reconstruction started, and comprehensive social and health reforms were scaled up [29, 44, 68, 69]. The country heavily invested in strengthening its health system through the implementation of community-based strategies, which continued to evolve and generate more support over time, investment in health infrastructure and health insurance, as well as integration with other programmes, such as MCH initiatives. In 2000, Rwanda developed a new malaria strategic plan, one of the first countries to have a strategic plan after the launch of the Roll Back Malaria partnership (RBM). The country introduced malaria community case management (HBM and subsequently iCCM) that achieved an increase of malaria case management. Collectively, with health systems innovations (CBHI and PBF), this laid the required groundwork for the national scale-up of critical malaria interventions (RDTs, LLINs, and ACT) beginning in 2006.

With strong national government support, appropriate policies, and financial resources from local and international sources, especially the Global Fund and PMI, Rwanda achieved significant progress against malaria in the first decade of the RBM era. NMCP’s innovative delivery of critical malaria control interventions has been heralded as a success story. In 2008, Rwanda was among the first countries to deploy ACT and RDTs through CHWs at the community level as well as one of the first countries to suspend IPTp with SP following SP resistance before the updated WHO policy recommendation on IPTp (October 2012) global guidance fight against malaria in Africa. Rwanda has also been a pioneer in developing and implementing IVM and resistance management strategies to prevent the emergence and spread of insecticide resistance, including by rotating insecticides every 2 years. Improved research capacity within the NMCP with support of partners has allowed the NMCP to monitor the durability and efficacy of critical malaria control interventions and hence identify low insecticide contents of LLINs distributed in 2012 and 2013. Factors such as the primary role of the Rwanda NMCP in defining and driving the malaria research agenda that generated local evidence to be used in the policy change process and capacity building of governance structures enabled all country-led innovation to persistent, internalized change and sustained better service delivery of malaria control interventions.

Access to appropriate malaria diagnosis and treatment for children through health facilities and CHWs increased, while LLIN coverage had reached near-universal coverage levels. Between 2005 and 2011, reductions of 85% in malaria incidence, 87% in outpatient malaria cases, 74% inpatient malaria deaths, and 71% malaria slide positivity rate was reported, while at the same time broader health system indicators improved dramatically [2, 3]. Compulsory diagnosis of malaria cases before treatment of malaria was included in the PBF indicators at health centres and community level, resulting in 99% of malaria cases being parasitological confirmed in 2015. PBF has been partially credited with increasing accountability, quality of care delivered by health workers, and an uptake of maternal and child health services [70].

Expansion of other malaria control interventions by CHWs, including IRS and community LLIN campaigns, have significantly improved coverage of malaria control interventions. The coverage of IRS in targeted areas reached over 98% in 2015 [5], while LLINs ownership and use increased to more than 70% between 2005 and 2015 (Additional file 3: Appendix 2: Fig. S2) [40, 71, 72]. As of February 2011, Rwanda was among the first African countries to achieve universal LLIN coverage.

Drug resistance monitoring also led the country to adopt different anti-malarial drugs, including artemisinin-based combinations (93–97%), that remain efficacious following recent studies carried out in 2018 [73].

The scale-up of health system strengthening interventions alongside malaria control interventions may have contributed to declines in malaria. Bucagu and colleagues [68] showed that increased coverage of maternal health services was associated with an increased capacity of the health workforce (both in numbers and skills), PBF, CBHI, and better leadership and governance. Besides, Rwanda’s “Ubudehe” programme provides an effective mechanism to identify those most in need of exemptions under the CBHI, enabling extensive coverage of health interventions, including malaria to the poor. Further research is needed to determine the impact of these changes on malaria outcomes.

An evaluation of the impact of malaria control on child mortality in Rwanda shows that the dramatic decline in child mortality that occurred in Rwanda from 1996–2000 to 2006–2010 coincided with a period of a rapid increase in malaria control interventions. Child mortality fell 61% during the evaluation period, and the prevalence of severe anaemia in children aged 6 to 23 months declined by 71%. These reductions in childhood morbidity and mortality were seen concurrently with a substantial increase in vector control activities. This supports the hypothesis that malaria control interventions contributed to the observed decline in child mortality in Rwanda from 2000 to 2010, even in the context of improving socioeconomic, maternal, and child health conditions.

### Challenges and implications for future control and elimination in Rwanda and elsewhere

The gains made through malaria control over the last century have been fragile in Rwanda, as shown in the dramatic increases in cases in the period starting 2012. Many factors have been incriminated in the country’s failure to sustain the reduction of malaria and progress toward elimination. These factors include climatic, environmental, technical, operational, and financial challenges, as well as factors related to human mobility, malaria parasites, and vectors, including resistance to drugs and insecticides.

As an example, malaria transmission has been on the rise in higher altitude areas, initially seen with malaria epidemics in the late 1980s at altitudes above 1500 m, and increases in temperature and rainfall during that time support the case for anthropogenic environmental changes extending the spatial limits of malaria [74]. Because there is little acquired immunity against malaria in these higher-altitude communities, outbreaks, and epidemics historically resulted in substantial morbidity and mortality in these areas [75]. A 1998 malaria epidemics in Byumba, a district with traditionally low transmission and high altitude of 2300 m, led to a fourfold increase in malaria admissions among pregnant women, and a fivefold increase in maternal malaria-attributable deaths [35]. Examples like this highlight the dangers of epidemics precipitated by relatively subtle climatic changes in areas of fringe or unstable transmission. Climatic changes have been shown to increase malaria in the last few years. Moreover, in recent decades, data show upticks in malaria transmission every two to 4 years, which could also be attributed to LLIN durability and procurement cycles.

Factors contributing to the volatility of malaria transmission in Rwanda also includes operational and governance challenges. For example, malaria elimination in Rwanda during GMEP, similar to other African countries, primarily failed due to time-limited, a decrease of funding, highly prescriptive and centralized programmes with low technical and operational capacity at the country level [67, 76]. Failure to sustain the GMEP at a global level led to a reduction in the capacity and resurgence of malaria in many countries, including Rwanda [77]. These programmes were run outside of national priorities systems and were designed internationally without building in-country capacity, including at sub-national levels, for proper planning and activity implementation. Similar factors have also arisen during the RBM era. In 2012 and 2015–2016, distributed LLINs were found to be substandard due to sub-optimal concentrations of insecticide contents, failing to meet WHO-required bio-efficacy standards for prequalification as later confirmed by the US Centers for Disease Control and Prevention [78]. LLIN durability monitoring also demonstrated that 22% and 50% of distributed LLINs had deteriorated within 6 and 12 months after distribution, respectively. Nearly 45% of LLINs required replacement after 24 months [79], which raised the possibility of shifting from a three-year LLIN distribution to a biennial distribution. In 2009 and 2012, delays in LLINs procurement and replacement in addition to the decline of LLIN efficacy, have been incriminated in the increase in malaria transmission in 2009 and 2012 [52]. Over the last 5 years, Rwanda has faced many delays in LLIN procurement and delivery not only due to late availability of funds and procurement processes issues but also due to insufficient Quality assurance (QA)/Quality Control (QC) processes for ensuring access to quality-assured malaria commodities due to poor quality LLINs deliveries [80].

Another example of factors contributing to historical upsurges in Rwanda is parasite resistance to anti-malarial drugs, as documented throughout the 1980s [24–26], and under investigation in recent years. K13 polymorphisms are infrequent but include variants associated with artemisinin resistance as shown by recent studies [81], whereby 222 *P. falciparum* isolates obtained from children in the Huye District of southern Rwanda from 2010 to 2015 and were sequenced to investigate the presence of *k13* polymorphisms. No polymorphisms were observed in 2010, but they were present in 2.5% and 4.5% of children in 2014 and 2015, respectively. In 2015, two isolates showed candidate *k13* resistance mutations (P574L and A675V), which are common in southeast Asia and associated with delayed parasite clearance. In the last 2 years, anti-malarial drug monitoring studies carried out in 3 sentinel sites although showing high efficacy of ACT (AL), have also found the highest proportion of artemisinin resistance-confirmed *k13* mutations reported (1–20% prevalence of *k13* 561H), a validated marker of artemisinin resistance, amongst 3 therapeutic efficacy studies (TES) sites in Rwanda [73]. This calls for continued monitoring and confirmation of suspected drug resistance in Rwanda.

Vector-related factors, including insecticide resistance and vector behavior change, may have also contributed to the increase of malaria in recent years. The resistance of *An. gambiae s.l.* to pyrethroids was confirmed in 2012 and to DDT and carbamates in 2013 [9]. In 2016, resistance to pyrethroids was established in 24 sites (75% of sentinel sites), and to DDT in 17 sites (53% of sentinel sites). Entomological monitoring has also shown a behavioral shift of malaria vectors from feeding indoors to outdoors as more than half of the sampled population (53–60%) has been found resting and biting outside. These high levels of insecticide resistance and vector behaviour changes may account for a reduction in the effectiveness of LLINs, the primary malaria vector control measure in Rwanda.

Failure to provide adequate and timely funding for key malaria interventions could also have played a part in recent upsurge. Increased funding in malaria control coincided with scaling up of malaria control interventions as seen in 2009 that allowed the country to procure LLINs for universal coverage in 2010–2011. However, subsequent years witnessed a decline and plateauing of malaria funding, resulting in prioritization of LLINs and IRS in high malaria endemic districts away from universal coverage as malaria transmission was on the rise.

Rwanda is not the only country in the region affected by an increase in the incidence of malaria as shown by the WHO WMR since 2013 [31, 82–84]. Therefore, additional risk factors for malaria transmission in Rwanda should be considered and further studied, including population movement between malaria-endemic and non-endemic regions both within Rwanda and across borders with neighboring countries. This increasing malaria transmission across the region threatens targets in the WHO Global Technical Strategy (GTS) for Malaria 2016–2030 and the Rwanda goal of malaria elimination. The history of malaria control in Rwanda offers valuable lessons about the supportive role of health system strengthening interventions, using local evidence, more nimble data, and robust monitoring of responses to drive malaria control. Besides, it calls for the need of a sustained and predictable financing and procurement to reverse current trends and achieve reductions in malaria toward national, regional, and global targets.

## Conclusion

Although the literature review provided useful information on the history of malaria control in Rwanda, one limitation in this study was the general scarcity of grey literature on malaria control interventions and epidemiology in Rwanda before the 1980s. Another limitation is the shortage of peer-reviewed published research in Rwanda from 1900 to 2018.

Rwanda was prosperous in reducing malaria transmission before and during the GMEP and again between 2005 and 2012, achieving significant reductions in this last period due to high coverage of effective malaria interventions and massive investment in health systems strengthening. Sustained implementation and massive scaling up of critical malaria control interventions have yielded fruitful results by reducing malaria in Rwanda. Adequate political and financial support with the integration of malaria interventions through a strengthened health system has allowed proper execution and quality delivery of effective malaria control interventions at decentralized levels. However, the history of malaria control in Rwanda demonstrates the fragility of these gains, most recently between 2012 and 2016. Several drivers are contributing to the changing malaria transmission in Rwanda, with an evolving relationship between humans, parasites, and vectors. Delays in funding for key malaria interventions such as LLINs affecting procurement as well as the purchase of substandard LLINs could also have played a part in the recent upsurges. Therefore, ensuring access to effective prevention and treatment on time remains a priority. Although Rwanda’s HMIS has improved considerably over time, there is a need for more rapid reporting as well as a systematic use of the data at all levels of decision making for a prompt response. Finally, political and financial supports should be maintained and even increased with innovative domestic financing. To provide further insights into the determinants of malaria progress in Rwanda in the past two decades, comprehensive empirical analysis of available data at subnational levels is needed. Also, as shown in Rwanda, country malaria control programmes should be dynamic and adaptive to respond and address changing contexts.

## Supplementary information

**Additional file 1: Appendix 1.** Literature search of peer-reviewed publications (Web of Knowledge, PubMed, Google Scholar, the World Health Organization Library (WHOLIS) and the Leopold Institute of Tropical Medicine (ITG)-Antwerp Library and grey literature (publicly available from the Rwanda NMCP and key partners/funding agencies) on malaria control in Rwanda between 1900 and 2019.

**Additional file 2: Appendix 2**. Rwanda Malaria Timeline.

**Additional file 3: Appendix 3.** Rwanda Mutligraph. Figure S1. Malaria cases and deaths, 1990–2018. Figure S2. ITN/LLIN ownership and use, 2005–2018. Figure 3. Community based health insurance coverage, 2002–2018. Figure S4. Malaria funding, 2005–2018.

## Data Availability

All critical malaria interventions coverage data analyzed during this study are included in this published article [and its Additional files 2, 2], are publicly available from the Institute of Statistics of Rwanda (http://statistics.gov.rw/datasource/demographic-and-health-survey-dhs) and the Demographic and Health Survey Program (https://www.dhsprogram.com/). The reported malaria cases and deaths data that support the findings of this study are available from the Health Information system of Rwandan Ministry of Health (MoH) and publicly available from the WHO World malaria reports (https://www.who.int/). All Rwandan MoH data, including community-based health insurance coverage, is available from the Rwanda MoH. Still, restrictions apply to the availability of these data, which were used under permission for the current study, and so are available per request to the Rwandan MoH.

## Abbreviations

ACT: Artemisinin-based combination therapy
AIDS: Acquired Immunodeficiency Syndrome
AL: Artemether-Lumefantrine
ANC: Antenatal care
AQ: Amodiaquine
Bti: *Bacillus thuringiensis israelensis*
CBHI: Community-based health insurance
CHWs: Community Healthcare Workers
CQ: Chloroquine
DDT: Dichloro-Diphenyl-Trichloroethane
DH: Demographic and Health Survey
EANMAT: East Africa Network for Monitoring Antimalarial Treatment
FANC: Focused antenatal care
GDP: Gross Domestic Product
Global Fund: The Global Fund to Fight AIDS, Tuberculosis, and Malaria
GMEP: Global Malaria Eradication Programme
HBM: Home-based management of fever
HIV: Human Immunodeficiency Virus
HMIS: Health Management Information System
iCCM: Integrated Community Case Management
IPTp: Intermittent Preventative Treatment in pregnancy
IRS: indoor residual spraying
ITG: Prince Leopold Institute of Tropical Medicine
ITNs: insecticide-treated nets
IVM: Integrated Vector Management
IWBF: Indigenous Well-Being Fund
KAP: Knowledge, attitudes, and practices
LLINs: Long-lasting insecticidal nets
MCP: malaria contingency plan
MIS: Malaria Indicator Survey
MoH: Ministry of Health
MPHSW: Ministry of Public Health and Social Work
NHA: National Health Account
NISR: National Institute of Statistics of Rwanda
NMCP: National Malaria Control Programme
PBF: Performance-based financing
PCR: Polymerase chain reaction
PI: *Plasmodium* index
PMI: President’s Malaria Initiative
PSI: Population Services International
QA: Quality assurance
QC: Quality control
RBM: Roll Back Malaria partnership
RDTs: Rapid diagnostic tests
SP: Sulfadoxine/Pyrimethamine
TES: Therapeutic efficacy studies
UN: United Nations
USAID: United States Agency for International Development
WHO: World Health Organization
WHOLIS: World Health Organization Library
WMR: World Malaria Report

## References

[1] WHO. World malaria report 2019. Geneva, World Health Organization, 2019. https://www.who.int/news-room/q-a-detail/world-malaria-report-2019.

[2] Karema C, Aregawi MW, Rukundo A, Kabayiza A, Mulindahabi M, Fall IS (2012). Trends in malaria cases, hospital admissions and deaths following scale-up of anti-malarial interventions, 2000–2010, Rwanda. Malar J..

[3] Otten M, Aregawi M, Were W, Karema C, Medin A, Bekele W (2009). Initial evidence of reduction of malaria cases and deaths in Rwanda and Ethiopia due to rapid scale-up of malaria prevention and treatment. Malar J..

[4] Sievers AC, Lewey J, Musafiri P, Franke MF, Bucyibaruta BJ, Stulac SN (2008). Reduced paediatric hospitalizations for malaria and febrile illness patterns following implementation of community-based malaria control programme in rural Rwanda. Malar J..

[5] PMI. PRESIDENT’S MALARIA INITIATIVE RWANDA Malaria Operational Plan FY 2018. 2017. https://www.pmi.gov/where-we-work/rwanda.

[6] Malaria OPDD, R. B. C. Ministry of Health. Rwanda Malaria Control Strategic Plan 2013–2018. Malaria & Other Parasitic Diseases Division-RBC/Ministry of Health (Rwanda), 2013. https://rbc.gov.rw/index.php?id=671.

[7] Malaria OPDD, R. B. C. Ministry of Health. Rwanda Malaria case management guidelines, 2015. 2015.

[8] Sinka ME, Bangs MJ, Manguin S, Coetzee M, Mbogo CM, Hemingway J (2010). The dominant Anopheles vectors of human malaria in Africa, Europe and the Middle East: occurrence data, distribution maps and bionomic precis. Parasit Vectors..

[9] Hakizimana E, Karema C, Munyakanage D, Iranzi G, Githure J, Tongren JE (2016). Susceptibility of *Anopheles gambiae* to insecticides used for malaria vector control in Rwanda. Malar J..

[10] Ivorra C. Sante et Maladies au Rwanda. In Paludisme. Bruxelles: AGCD; 1982; pp. 427–447.

[11] Le Mattlet G (1935). Kapfura ou le Kaffindo-findo..

[12] Munyantore S (1989). Historique de la lutte antipaludique au Rwanda. Rev Med Rwandaise..

[13] Meyus H, Lips M, Caubergh H (1962). The current state of the problem of highland malaria in Ruanda-Urundi. Ann Soc Belge Med Trop..

[14] Schwetz J (1941). Contribution à l’étude des Anopheles du Congo oriental (Lac Kivu, Lac Albert). Ann Soc Belge Med Trop..

[15] Van Poucke G (1979). A comparative study on the use of intramuscular chloroquine and quinine (as Quinimax) in the treatment of uncomplicated falciparum malaria in adults. East Afr Med J.

[16] Sambo L (2011). Health systems and primary health care in the African region. Afr Health Monitor..

[17] Schwetz J. Recherche sur le paludisme dans les villages et les camps de la division du Mwoghwalu des mines d’or de Kilo (Congo Belge). Institut Colonial Belge. 1940; Memoires in 8eme.

[18] Jadin J, Fain A (1951). Contribution to the study of malaria in high altitudes. Ann Soc Belge Med Trop..

[19] Chardome M, Peel E, Lambrecht FL (1953). Malaria in the Ruzizi valley. Ann Soc Belge Med Trop..

[20] Vincke IH, Jadin JB (1911). Contribution a l’etude de l’anophelisme en pays d’altitude. Ann Soc Belge Med Trop..

[21] Jadin JB (1952). Rapport sur la campagne de dedetisation dans le territoire d’Astrida. Ann Soc Belge Med Trop..

[22] Ettling MB, Shepard DS (1991). Economic cost of malaria in Rwanda. Trop Med Parasitol..

[23] Mafart Y, Pieron R, Melchior JC (1981). [A new case of *Plasmodium falciparum* malaria resistance to chloroquine in Africa (Rwanda)](in French). Med Trop (Mars)..

[24] Charmot G, Amat-Roze JM, Rodhain F, Le Bras J, Coulaud JP (1991). Geographic approach to the epidemiology of chloroquine-resistance of *Plasmodium falciparum* in tropical Africa. Ann Soc Belge Med Trop..

[25] Giboda M, Vanista J, Dastych P (1988). The first report of *Plasmodium falciparum* resistant to chloroquine plus sulphadoxine/pyrimethamine in Rwanda. Trans R Soc Trop Med Hyg.

[26] Gascon J, Soldevila M, Merlos A, Bada JL (1985). Chloroquine and amodiaquine resistant falciparum malaria in Rwanda. Lancet.

[27] Gascon J, Pluymaekaers J, Bada JL (1986). Chloroquine-resistant falciparum malaria from Rwanda. Trans R Soc Trop Med Hyg.

[28] Allen S, Van de Perre P, Serufilira A, Lepage P, Carael M, DeClercq A (1991). Human immunodeficiency virus and malaria in a representative sample of childbearing women in Kigali, Rwanda. J Infect Dis..

[29] Binagwaho A, Farmer PE, Nsanzimana S, Karema C, Gasana M, de Dieu Ngirabega J (2014). Rwanda 20 years on: investing in life. Lancet.

[30] Musafili A, Essen B, Baribwira C, Binagwaho A, Persson L-A, Selling KE (2015). Trends and social differentials in child mortality in Rwanda 1990–2010: results from three demographic and health surveys. J Epidemiol Community Health.

[31] WHO. World Malaria Report 2014. Geneva, World Health Organization, 2014. https://www.who.int/malaria/publications/world_malaria_report_2014/en/.

[32] Overseas Development Institute. Overseas Development Institute annual report 2010–2011. Overseas Development Institute, 2012.

[33] Hodes RM, Wolday D, Kibreab T (1997). Sensitivities of malaria in Zaire. Trop Doct.

[34] Cooperation Belge au developpement. evaluation de l’appui du Prgramme integre de lutte contre le Paludisme au Rwanda1994–2006. 2008.

[35] Hammerich A, Campbell OMR, Chandramohan D (2002). Unstable malaria transmission and maternal mortality–experiences from Rwanda. Trop Med Int Health..

[36] Rwanda Ministry of Health. Rwanda MoH annual report 2000. 2001. https://moh.gov.rw/index.php?id=512.

[37] Rwagacondo CE, Karema C, Mugisha V, Erhart A, Dujardin JC, Overmeir CV (2004). Is amodiaquine failing in Rwanda? Efficacy of amodiaquine alone and combined with artesunate in children with uncomplicated malaria. Trop Med Int Health..

[38] Rwagacondo CE, Niyitegeka F, Sarushi J, Karema C, Mugisha V, Dujardin JC (2003). Efficacy of amodiaquine alone and combined with sulfadoxine-pyrimethamine and of sulfadoxine pyrimethamine combined with artesunate. Am J Trop Med Hyg.

[39] EANMAT (2001). Monitoring antimalarial drug resistance within National Malaria Control Programmes: the EANMAT experience. Trop Med Int Health..

[40] Rwanda National Institute of Statistics of Rwanda (NISR) & ORC Macro. Rwanda Demographic and Health Survey 2005. 2006. https://www.statistics.gov.rw/publication/rwanda-demographic-and-health-survey-report-dhs-2005.

[41] Van Geertruyden J-P, Ntakirutimana D, Erhart A, Rwagacondo C, Kabano A, D’Alessandro U (2005). Malaria infection among pregnant women attending antenatal clinics in six Rwandan districts. Trop Med Int Health..

[42] Mugeni C, Levine AC, Munyaneza RM, Mulindahabi E, Cockrell HC, Glavis-Bloom J (2014). Nationwide implementation of integrated community case management of childhood illness in Rwanda. Glob health, Sci Pract..

[43] WHO. Scaling up home-based management of malaria: from research to implementation. Geneva, World Health Organization, 2004. https://www.who.int/malaria/publications/atoz/who_htm_mal_2004_1096/en/.

[44] Logie DE, Rowson M, Ndagije F (2008). Innovations in Rwanda’s health system: looking to the future. Lancet.

[45] Kalk A, Paul FA, Grabosch E (2010). ‘Paying for performance’in Rwanda: does it pay off?. Trop Med Int Health..

[46] Schmidt JO, Mayindo JK, Kalk A (2006). Thresholds for health insurance in Rwanda: who should pay how much?. Trop Med Int Health..

[47] Overseas Development Institute- Development progress. Rwanda’s progress in health: Leadership, performance and insurance. In Development Progress, Overseas Development Institute. London, UK2011. pp. 1–38.

[48] Nisr M, Macro O (2012). USARwanda demographic and health survey 2010.

[49] Malaria OPDD, R. B. C. Ministry of Health, Jhpiego Rwanda. Malaria prevalence iamong pregnant Rwandan women—Study report. 2014.

[50] Rwanda Ministry of Health. Rwanda Malaria programme performance review. Kigali-Rwanda, Rwanda Ministry of Health, 2017. https://moh.gov.rw/index.php?id=512.

[51] PMI. President’s Malaria Initiative Rwanda Malaria Operational Plan FY 2014. 2013. https://www.pmi.gov/where-we-work/rwanda.

[52] PMI. President’s Malaria Initiative Malaria Operational Plan (MOP) Rwanda FY 2016. Washington, DC2016. https://www.pmi.gov/where-we-work/rwanda.

[53] Malaria opDD, R. B. C. Rwanda annual malaria report 2015. 2015.

[54] Barat L, Schubert J. External Evaluation of the Pilot Phase of the Home-Based Management of Malaria Program in Rwanda, Final Report. Kigali: USAID/BASICS, USAID/RPM Plus, Integrated National Malaria Control Program, Rwanda. 2007.

[55] Franco C, Schubert J, Yameogo M, Briggs J, Kabuya W, Hitayezu F (2008). Evaluation of the Home Based Management of Malaria Strategy in Rwanda: 2008.

[56] Rwanda Ministry of Health. National Community Health Strategic Plan 2013-2018. 2013. https://moh.gov.rw/index.php?id=512.

[57] Karema C, Imwong M, Fanello CI, Stepniewska K, Uwimana A, Nakeesathit S (2010). Molecular correlates of high-level antifolate resistance in Rwandan children with *Plasmodium falciparum* malaria. Antimicrob Agents Chemother.

[58] Fanello CI, Karema C, Avellino P, Bancone G, Uwimana A, Lee SJ (2008). High risk of severe anaemia after chlorproguanil-dapsone + artesunate antimalarial treatment in patients with G6PD (A-) deficiency. PLoS ONE.

[59] Fanello CI, Karema C, van Doren W, Rwagacondo CE, D’Alessandro U (2006). Tolerability of amodiaquine and sulphadoxine-pyrimethamine, alone or in combination for the treatment of uncomplicated *Plasmodium falciparum* malaria in Rwandan adults. Trop Med Int Health..

[60] Rwanda Ministry of Health. National Health account Rwanda 2006 with HIV/AIDs, Malaria and Reproductive health subaccounts. Kigali-Rwanda. 2008. https://moh.gov.rw/index.php?id=512.

[61] Rwanda Ministry of Health. National Health account Rwanda 2008. Kigali-Rwanda. 2012. https://moh.gov.rw/index.php?id=512.

[62] Rwanda Ministry of Health. Rwanda MoH Annual Report 2012. 2013. https://moh.gov.rw/index.php?id=512.

[63] Clinton Health Access Initiative. Maintaining the Gains in Global Malaria Control. 2013.

[64] National Institute of Statistics of Rwanda (NISR) [Rwanda], MoHMR, International aI. Rwanda Demographic and Health Survey 2014–15. ICF International edition. pp. 513. 530 Gaither Road, Suite 500, Rockville, MD 20850, USA, National Institute of Statistics of Rwanda (NISR) [Rwanda], Ministry of Health (MOH) [Rwanda], and ICF International. 2015, 2016,513. https://www.dhsprogram.com/what-we-do/survey/survey-display-468.cfm.

[65] Rwanda Ministry of Health. Rwanda Malaria contingency plan 2015. 2015. https://moh.gov.rw/index.php?id=512.

[66] Sievers AC, Lewey J, Musafiri P, Franke MF, Bucyibaruta BJ, Stulac SN (2008). Reduced paediatric hospitalizations for malaria and febrile illness patterns following implementation of a community-based malaria control programme in rural Rwanda. World Hosp Health Serv..

[67] Mendis K, Rietveld A, Warsame M, Bosman A, Greenwood B, Wernsdorfer WH (2009). From malaria control to eradication: the WHO perspective. Trop Med Int Health..

[68] Bucagu M, Kagubare JM, Basinga P, Ngabo F, Timmons BK, Lee AC (2012). Impact of health systems strengthening on coverage of maternal health services in Rwanda, 2000–2010: a systematic review. Reprod Health Matters..

[69] Sayinzoga F, Bijlmakers L (2016). Drivers of improved health sector performance in Rwanda: a qualitative view from within. BMC Health Serv Res..

[70] Sekabaraga C, Diop F, Soucat A (2011). Can innovative health financing policies increase access to MDG-related services?. Evidence from Rwanda. Health Policy Plan..

[71] National Institute of Statistics of RRMo, Health and ICF International,. Rwanda DHS 2014–15: Key Indicators. DHS Macro, 2015.

[72] National Institute of Statistics of RMoH, ICF. International. Rwanda Demographic and Health Survey 2010. Measure DHS, ICF Macro, 2012. https://www.dhsprogram.com/what-we-do/survey/survey-display-468.cfm.

[73] Uwimana A, Legrand E, Stokes BH, Ndikumana JM, Warsame M, Umulisa N (2020). Emergence and clonal expansion of in vitro artemisinin-resistant *Plasmodium falciparum* kelch13 R561H mutant parasites in Rwanda. Nat Med..

[74] Loevinsohn ME (1994). Climatic warming and increased malaria incidence in Rwanda. Lancet.

[75] Lindsay S, Zetocha. A feasible approach for implementing greater levels of satellite autonomy. In AIAA Defense and Civil Space Programs Conference and Exhibit. 1998: 5232.

[76] Trigg PI, Kondrashin AV (1998). Commentary: malaria control in the 1990s. Bull World Health Organ.

[77] WHO. Re-examination of the global strategy of malaria eradication, Provisionnal Agenda Item 2.4, 22nd World Health Assembly. Geneva, World Health Organization, 1969. https://apps.who.int/iris/handle/10665/144292?show=full.

[78] Binagwaho A, Freeman R, Scott K, Harward S, Mulindahabi M, Karema C. Improving international accountability—a tool for protecting health as a basic human right. Health Hum Rights. April 2015. Accessed 20 August 2015.

[79] Hakizimana E, Cyubahiro B, Rukundo A, Kabayiza A, Mutabazi A, Beach R (2014). Monitoring long-lasting insecticidal net (LLIN) durability to validate net serviceable life assumptions, in Rwanda. Malar J..

[80] Malaria OPDD, R. B. C. Ministry of Health,. Rwanda Malaria Program Review 2019. RBC, 2019.

[81] Constanza T, PranbanjaN PG, Bayingana C, Sifft KC, Geus D, Ndoli J (2016). Artemisinin resistance–associated K13 polymorphisms of *Plasmodium falciparum* in Southern Rwanda, 2010–2015. Am J Trop Med Hyg.

[82] WHO. World Malaria Report 2016. Geneva, World Health Organization, 2016. https://www.who.int/malaria/publications/world-malaria-report-2016/report/en/.

[83] WHO. World Malaria report 2017. Geneva, World Health Organization, 2017. https://www.who.int/malaria/publications/world-malaria-report-2017/en/.

[84] WHO. World Malaria Report 2018. Geneva, World Health Organization, 2018. https://www.who.int/campaigns/malaria-day/2018/en/2002–2018.

