# Supplementary material for: History of malaria control in Rwanda: implications for future elimination in Rwanda and other malaria-endemic countries
Source: Malar J. 2020 Oct 7;19:356. doi: 10.1186/s12936-020-03407-1 (PMC7539391; doi:10.1186/s12936-020-03407-1)
Supplement: Supplementary file 2 — Additional file 2: Appendix 2. Rwanda Malaria Timeline. [file 12936_2020_3407_MOESM2_ESM.pdf]

Period 1: First documentation of malaria to the development of the GMEP (1900–1954)

- **1917** First documentation of malaria cases
- **1920–1930** Report of increase of malaria cases
- **1930–1940** Use of quinine for malaria treatment; Parasite and vector surveys by the Medical Laboratory of Ruanda-Urundi
- **1946** New malaria control initiatives with a focus on environmental management
- **1949–1951** DDT spraying campaigns in Butare (prior to GMEP)
- **1952–1954** DDT spraying campaigns expansion to Usumbura & Bubanza in 1952 and Shangungu in 1954 (prior to GMEP)

Period 2: The Global Malaria Eradication Program (GMEP) in Rwanda (1955–1969)

- **1955–1960** GMEP with DDT targeted spraying campaigns in Ruanda-Urundi
- **1962** First malaria stratification by Meyus; End of GMEP activities in Rwanda; Severe Epidemics of malaria in Byumba and Ruhengeri
- **1969** End of GMEP

Period 3: Post GMEP to the Genocide against the Tutsi (1970–1994)

- **1980** Treatment standardization; Start of drug resistance monitoring system
- **1981** Report of earliest therapeutic failures of chloroquine treatment for *P. falciparum* in Rwanda
- **1982** Updated malaria stratification by Yvorry Canon
- **1985** Report of amodiaquine and sulfadoxine pyrimethamine drug resistance
- **1989** Establishment of the National Malaria Control Program
- **1994** 1994 Genocide against the Tutsi killing > 1 million people followed by malaria epidemics

Period 4: Reestablishment of malaria control (1995–2005)

- **1995** Development of first malaria policy
- **1995** Spike in malaria incidence plus massive population movement (estimated 1.4 Million internally displaced and 1.5 Millions returnees)
- **1996** Introduction of mosquito nets in the private sector
- **1998–2004** Severe epidemics of malaria
- **1999–2000** Community based health insurance (CBHI) pilot in 3 districts
- **2000** Pilot distribution of 40000 mosquito nets for project evaluation in Kayonza
- **2000** Comprehensive social and health reforms with establishment of CBHI and initial decentralization of public health
- **2000** Introduction of CQ weekly doses for prevention of malaria in pregnancy + iron supplementation in standard ANC care
- **2001** Adoption of AQ+SP for malaria treatment; Introduction of Performance based Financing (PBF) and Ubudehe programme
- **2004** HBM pilot in 6 districts with AQ+SP; Roll out of PBF at health facilities; Scale up of Ubudehe programme countrywide
- **2005** Start of GFATM support for malaria; Scale up of IPTp and LLINs for routine distribution (EPI and ANC)

Period 5: Current malaria response in Rwanda (2006–2018)

- **2006** First countrywide mass LLIN campaign for children under five, National rollout of CBHI introduction of ACTs; Reform of public health decentralization
- **2007** Start of PMI support in Rwanda; Scale up of HBM with ACTs to 9 districts; IRS using pyrethroids in 3 districts
- **2008** Introduction of RDTs at community level Scale up of ACTs countrywide, including in the private sector; Discontinuation of IPTp
- **2009** Start of iCCM implementation (Malaria, pneumonia, diarrhea); First countrywide mass LLIN campaign for all ages and households
- **2010** Universal coverage of LLINs and malaria diagnostic achieved
- **2011** IVM policy; Scale up of IRS using pyrethroids in 5 districts; Rollout of PBF at the community level for CHWs
- **2012** Distribution of substandard LLINs
- **2013–2014** Replacement of substandard LLINs; Targeted LLINs in high malaria burden districts and Switch to carbamates for IRS
- **2014** Switch to organophosphates for IRS; Targeted LLINs in high malaria burden districts
- **2015** Development of malaria contingency plan; Targeted LLINs in high malaria burden districts
- **2015** Pilot use of biological larviciding
- **2016** Switch to organophosphate for IRS and expansion of community case management of malaria to all age groups (RDTs and ACTs)
