# Supplementary material for: History of malaria control in Rwanda: implications for future elimination in Rwanda and other malaria-endemic countries
Source: Malar J. 2020 Oct 7;19:356. doi: 10.1186/s12936-020-03407-1 (PMC7539391; doi:10.1186/s12936-020-03407-1)
Supplement: Supplementary file 3 — Additional file 3: Appendix 3. Rwanda Mutligraph. Figure S1. Malaria cases and deaths, 1990–2018. Figure S2. ITN/LLIN ownership and use, 2005–2018. Figure 3. Community based health insurance coverage, 2002–2018. Figure S4. Malaria funding, 2005–2018. [file 12936_2020_3407_MOESM3_ESM.pdf]

Figure 1: Malaria cases and deaths, 1990–2018

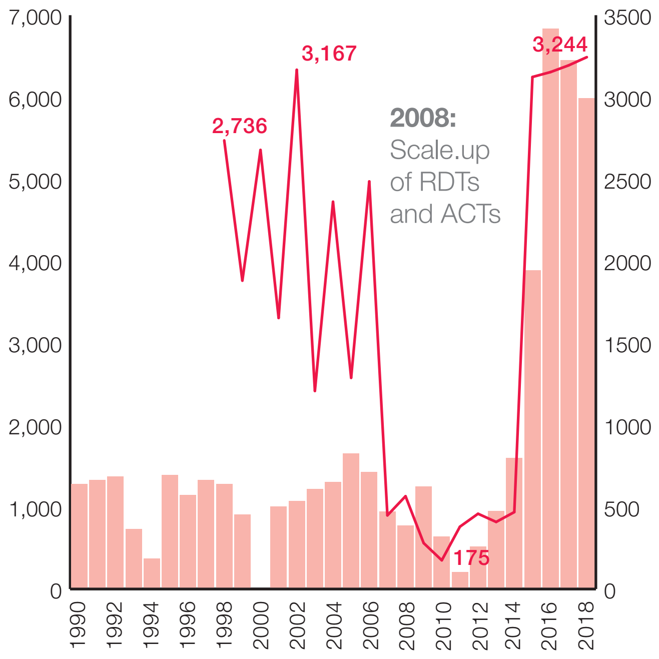

Presumed and confirmed malaria cases, in thousands (HMIS & WHO WMR) *(left y-axis)*

Reported malaria deaths (WHO WMR) (*right y-axis*)

Figure 2: ITN/LLIN ownership and use, 2005–2018

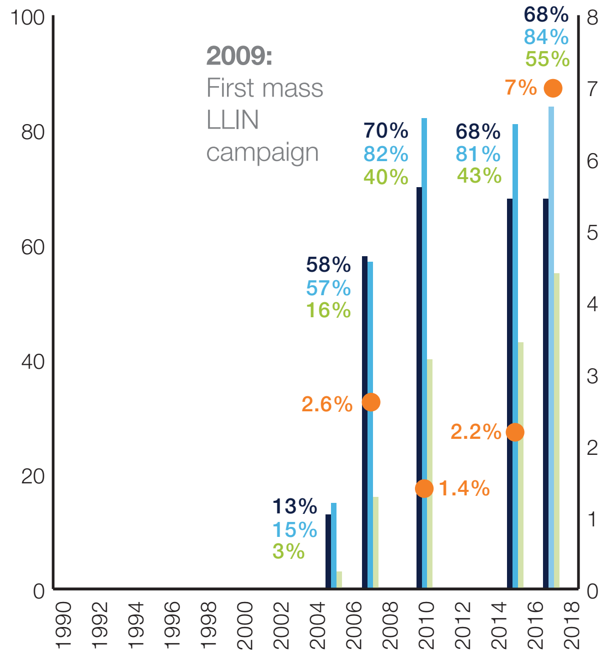

ITN usage (% of children under five who slept under an ITN the previous night) (DHS) *(left axis)*

ITN ownership (% households with at least one ITN) (DHS) *(left axis)*

ITN ownership (% households with at least one ITN for every two people) (DHS) (*left axis*)

% prevalence of malaria in children 6–59 months (DHS) (*right axis*)

Figure 3: Community based health insurance coverage, 2002–2018

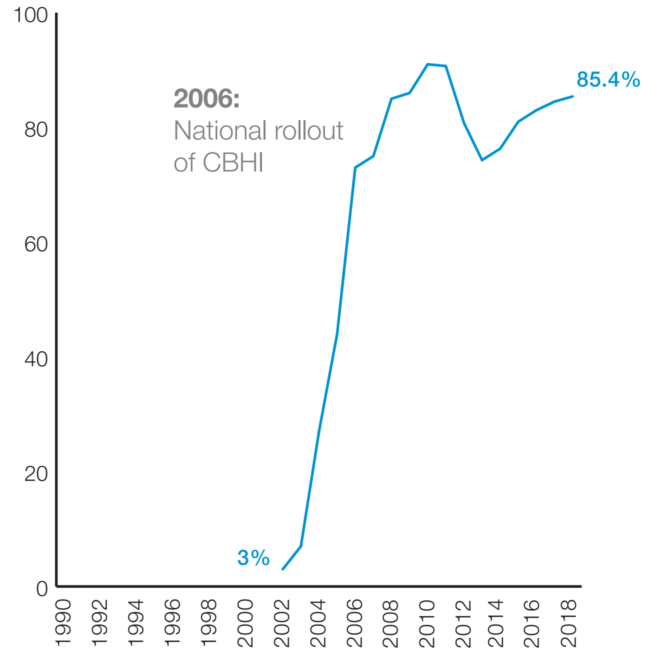

Coverage of community-based health insurance (CBHI) (%) (MoH)

Figure 4: Malaria funding, 2005–2018

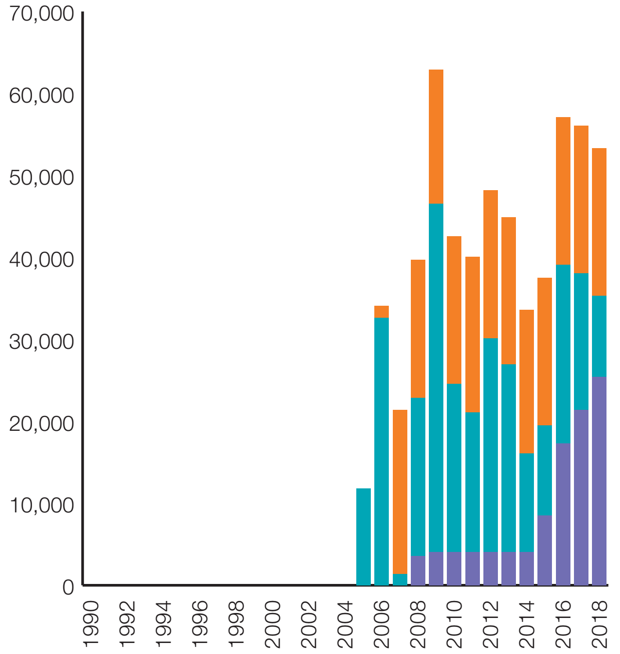

Domestic government malaria funding in millions $USD

Global Fund malaria funding in millions $USD (Global Fund Data Explorer)

US President's Malaria Initiative malaria funding in millions $USD (PMI Resource Library)
